# Artificial intelligence for image recognition in diagnosing oral and oropharyngeal cancer and leukoplakia

**DOI:** 10.1038/s41598-025-85920-4

**Published:** 2025-01-29

**Authors:** Benedikt Schmidl, Tobias Hütten, Steffi Pigorsch, Fabian Stögbauer, Cosima C. Hoch, Timon Hussain, Barbara Wollenberg, Markus Wirth

**Affiliations:** 1 Department of Otolaryngology Head and Neck Surgery, Technical University Munich, Munich, Germany; 2 Department of RadioOncology, Technical University Munich, Munich, Germany; 3 Institute of Pathology, Technical University Munich, Munich, Germany

**Keywords:** Image recognition, OSCC, ChatGPT, OPSCC, Artificial Intelligence, Cancer, Surgical oncology

## Abstract

Visual diagnosis is one of the key features of squamous cell carcinoma of the oral cavity (OSCC) and oropharynx (OPSCC), both subsets of head and neck squamous cell carcinoma (HNSCC) with a heterogeneous clinical appearance. Advancements in artificial intelligence led to Image recognition being introduced recently into large language models (LLMs) such as ChatGPT 4.0. This exploratory study, for the first time, evaluated the application of image recognition by ChatGPT to diagnose squamous cell carcinoma and leukoplakia based on clinical images, with images without any lesion as a control group. A total of 45 clinical images were analyzed, comprising 15 cases each of SCC, leukoplakia, and non-lesion images. ChatGPT 4.0 was tasked with providing the most likely diagnosis based on these images in scenario one. In scenario two the image and the clinical history were provided, whereas in scenario three only the clinical history was given. The results and the accuracy of the LLM were rated by two independent reviewers and the overall performance was evaluated using the modified Artificial Intelligence Performance Index (AIPI. In this study, ChatGPT 4.0 demonstrated the ability to correctly identify leukoplakia cases using image recognition alone, while the ability to diagnose SCC was insufficient, but improved by including the clinical history in the prompt. Providing only the clinical history resulted in a misclassification of most leukoplakia and some SCC cases. Oral cavity lesions were more likely to be diagnosed correctly. In this exploratory study of 45 images of oral lesions, ChatGPT 4.0 demonstrated a convincing performance for detecting SCC only when the clinical history was added, whereas Leukoplakia was detected solely by image recognition. ChatGPT is therefore currently insufficient for reliable OPSCC and OSCC diagnosis, but further technological advancements may pave the way for the use in the clinical setting.

## Introduction

Head and neck squamous cell carcinoma (HNSCC) is a heterogenous disease, that derives from the mucosal epithelium in the oral cavity, pharynx and larynx^1^. Although the oral cavity and oropharynx can be accessed and visualized easily, the majority of HNSCC are diagnosed at advanced stages. With a rising incidence of oropharyngeal squamous cell carcinoma (OPSCC), due to an association with the human papillomavirus Virus (HPV) and a long latency between infection with HPV and the development of HPV + HNSCC, it is estimated that the effect of an HPV vaccination will not be reflected in HNSCC prevalence until 2060^2^. Despite better survival rates for patients with HPV-associated OPSCC compared to HPV-negative cases, there is an urgent need for strategies to enable earlier diagnosis and prevention. Currently, no standardized or validated screening tools exist for patients at risk of oral cavity or oropharyngeal cancer, although liquid biopsy techniques such as cell-free human papillomavirus DNA^3^, thrombocytes^4^, and many others^5^ are investigated.

Early-stage cancers are often asymptomatic and can mimic benign conditions, decreasing the likelihood that affected individuals will seek medical attention. Screening programs therefore offer a critical opportunity for early detection^6^. In addition, premalignant lesions (OPLs), including leukoplakia, erythroplakia and dysplastic leukoplakia, as well as tumor recurrence are also accessible to visual inspection^7,8^. One of the first randomized control studies for a population based oral cancer screening was performed in Kerala, India, where 87,655 patients at risk for oral cancer were screened over a 15-year period. This led to a reduction in the incidence of oral cancer and a significant 34% reduction in oral cancer mortality, highlighting the potential benefit of a population-based screening^9^. However, limitations in resources, costs, time, and availability of healthcare workers necessitate innovative strategies to overcome these challenges.

One of these strategies is the implementation of artificial intelligence. Artificial intelligence (AI), in the form of deep learning (DL) and natural language processing (NLP), have enabled the development of large language models (LLMs) like Generative Pre-trained Transformers (GPT)^10,11^. These models access large datasets in a short amount of time and are able to gather the information of recent studies, but also historical data that can be summarized as the basis of a modern approach to discuss oncological cases^12–14^, including head and neck cancer^15,16^. The ability of AI to organize and structure data presents opportunities for these tools to assist, or even guide, clinical decisions^17,18^. One of the most recent features of ChatGPT 4.0 are voice and image recognition capabilities that promise to expand the use of these tools in the current healthcare landscape, with applications including the identification of abnormalities in medical images or skin lesions^19,20^. Since oral cavity and most oropharyngeal lesions derive from the mucosal epithelium and an image can be easily and non-invasively obtained in most cases, the image recognition of ChatGPT might also detect squamous cell carcinoma or pre-malignant lesions. This ability could then be used as a screening tool or for the tumor follow up after an initial malignancy of the head and neck^21^.

The primary objective of this exploratory study was to assess the LLMs ability to differentiate between SCC, premalignant lesions, and benign or lesion-free conditions, as well as to propose next diagnostic steps. In a second step the clinical history of the patients was added to simulate a realistic clinical setting. Two independent reviewers assessed the results, and the overall performance was analyzed to provide insights into the potential role of image interpretation by ChatGPT 4.0 in clinical practice.

## Materials and methods

### Patient cohort

This study included clinical images of oral and oropharyngeal squamous cell carcinoma patients, images of leukoplakia and images without an oral pathology. The images were always used in a fully anonymized way, any detail that might allow the identification of a patient was removed. The electronic patient file and MDT documents provided clinical and histological tumor characteristics and patient ages at the time of diagnosis. This study comprised a total of 45 patients at the department of otorhinolaryngology/head and neck surgery, Klinikum rechts der Isar, Technical University of Munich. The patients’ ages spanned from 49 to 88 years. 25 images in this study showed squamous cell carcinoma, while two of these images depicted oropharyngeal lesions. Another 15 images showed oral leukoplakia, and another 15 images showed no lesion in the oral cavity or oropharynx (two cases). These were used as a control mechanism. All images were taken by a single investigator and sourced from a controlled image database where imaging protocols were consistent. The images also needed to clearly show the lesion or lesion-free area without obstructions and there was no pre-processing. Before analysis, the dataset was reviewed by another independent head and neck surgeon to confirm that all images met the study’s quality criteria. To ensure patient confidentiality, the data were anonymized before being shared with the researchers, making patient identification impossible. This study was approved by the ethics committee of the Technical University of Munich. The characteristics of the patient cohort are depicted in the Supplementary Table 1. Informed consent was obtained from all subjects and/or their legal guardian(s).

### Artificial Intelligence/Image recognition by ChatGPT prompt formatting and data evaluation

**Fig. 1:**
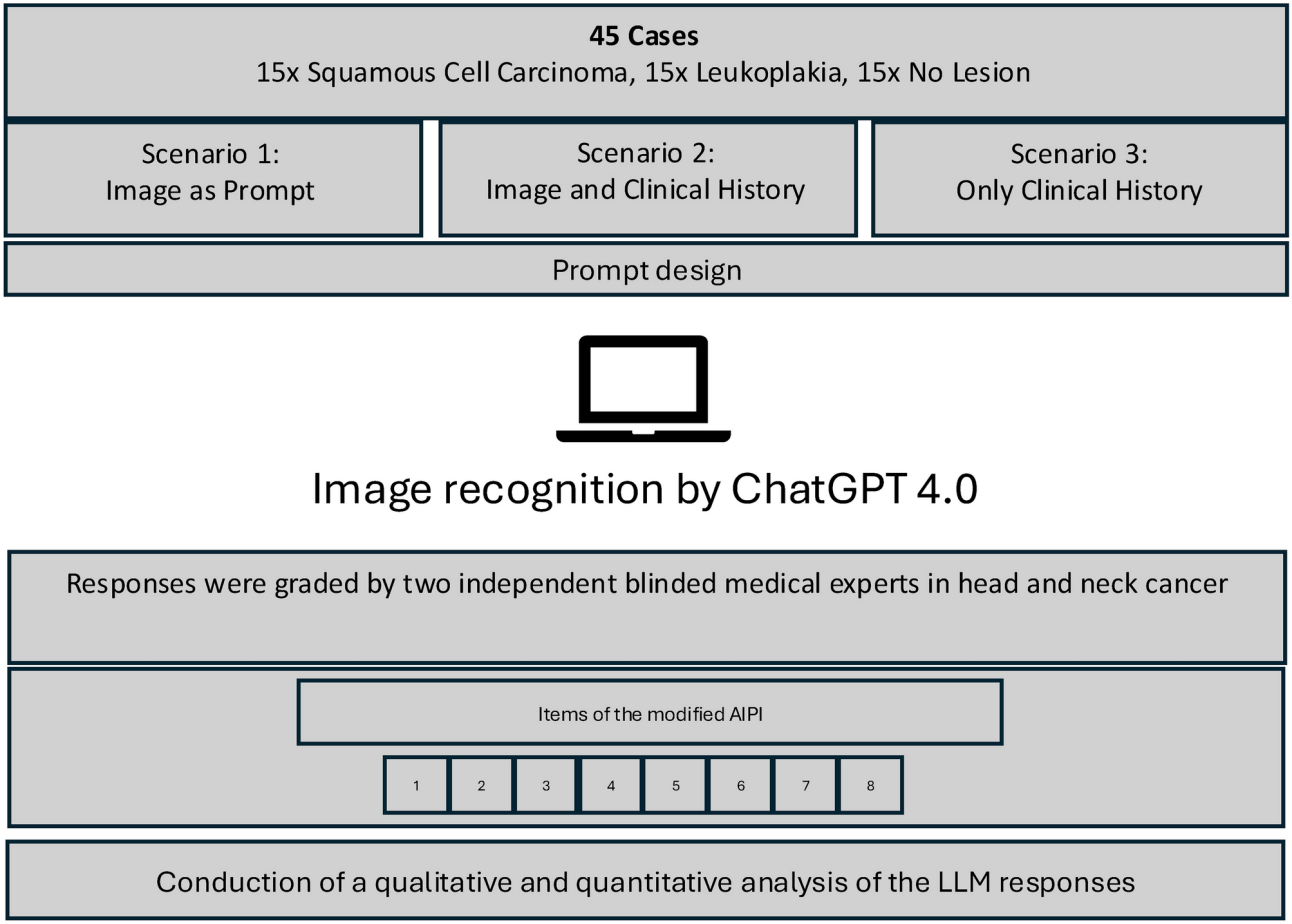
Flowchart of overall study design. Depiction of the workflow of the study including the grading of responses by image recognition by ChatGPT 4.0. Evaluation of the responses by two independent reviewers.

ChatGPT 4.0 is an AI-powered chatbot that is accessible to the public. Chatbots use transformer-based language models to generate human-like text responses. The interaction is most of the time based on users submitting questions (prompts) through a website interface. The LLMs are then able to analyze the contextual relationships between the words in the user’s query to formulate a response**.** Image analysis is a recently added feature of ChatGPT and is tested in this study. The study design and is shown in a flowchart in Fig. 1.

The prerequisite of studies assessing the use of an LLM is rigorous prompt testing. Therefore, eight different versions of the prompt were tested prior to selecting the most appropriate prompt for this study. This standardized prompt format was employed to input the patient’s clinical history into ChatGPT in addition to the clinical image. The prompt was as follows: “A patient presents with this intraoral lesion. What is the most likely diagnosis and differential diagnosis? What would be the next diagnostic steps?”. When the clinical history was used the prompt was “The patient presents with (XX). The patient is (XX) drinking and (XX) smoking. The patient additionally has (XX). The patient sent this picture and wants to know the most likely diagnosis. What are the next diagnostic steps?”. The following scenario could be presented: " The patient presents with dysphagia for 4 months, night sweats. The patient is rarely drinking and active, 25py smoking. The patient additionally has no comorbidities. The patient sent this picture and wants to know the most likely diagnosis. What are the next diagnostic steps?”. Each prompt was used for 3 times to get the most consistent answer. No further interaction was initiated after the response. The LLMs prompt history was erased, and the next question was asked. To prevent any influence from previous responses, a new session was initiated for each prompt. To guarantee a most consistent result, a subset of prompts was also used in an additional part of this study in three different web browsers, at three different times of the day and on three different days to guarantee a most consistent result (Supplementary Fig. 2). The responses were collected, and in a double-blind method evaluated. The two independent reviewers got the image and the response and were uninformed about which AI model stated the response. All reviewers independently scored the answers to mitigate subjective biases. The answers provided by ChatGPT 4.0 were assessed using the grading scales of the Artificial Intelligence Performance Index (AIPI) as proposed by Lechien et al.^22^. In addition, the category of image description was implemented with the four grades of 0 = Absence of image description/Referral, 1 = Mentioning of the image/Referral, 2 = General description, 3 = Image recognition and location. Each suspected diagnosis generated by ChatGPT, whether based solely on image recognition or supplemented with clinical history, was compared to the confirmed histological diagnosis. This approach ensured an objective and accurate assessment of ChatGPT’s ability to identify squamous cell carcinoma, leukoplakia, and benign or lesion-free conditions. Cohen’s kappa coefficient was used to measure inter-rater reliability. For the responses of the LLM the Mann–Whitney U test was used to identify significant differences. When multiple hypothesis tests were conducted, P-values were adjusted using the Bonferroni correction method. A p-value of less than 0.05 was considered statistically significant. The dataset is depicted in the Supplementary Material.

## Results

**Fig. 2:**
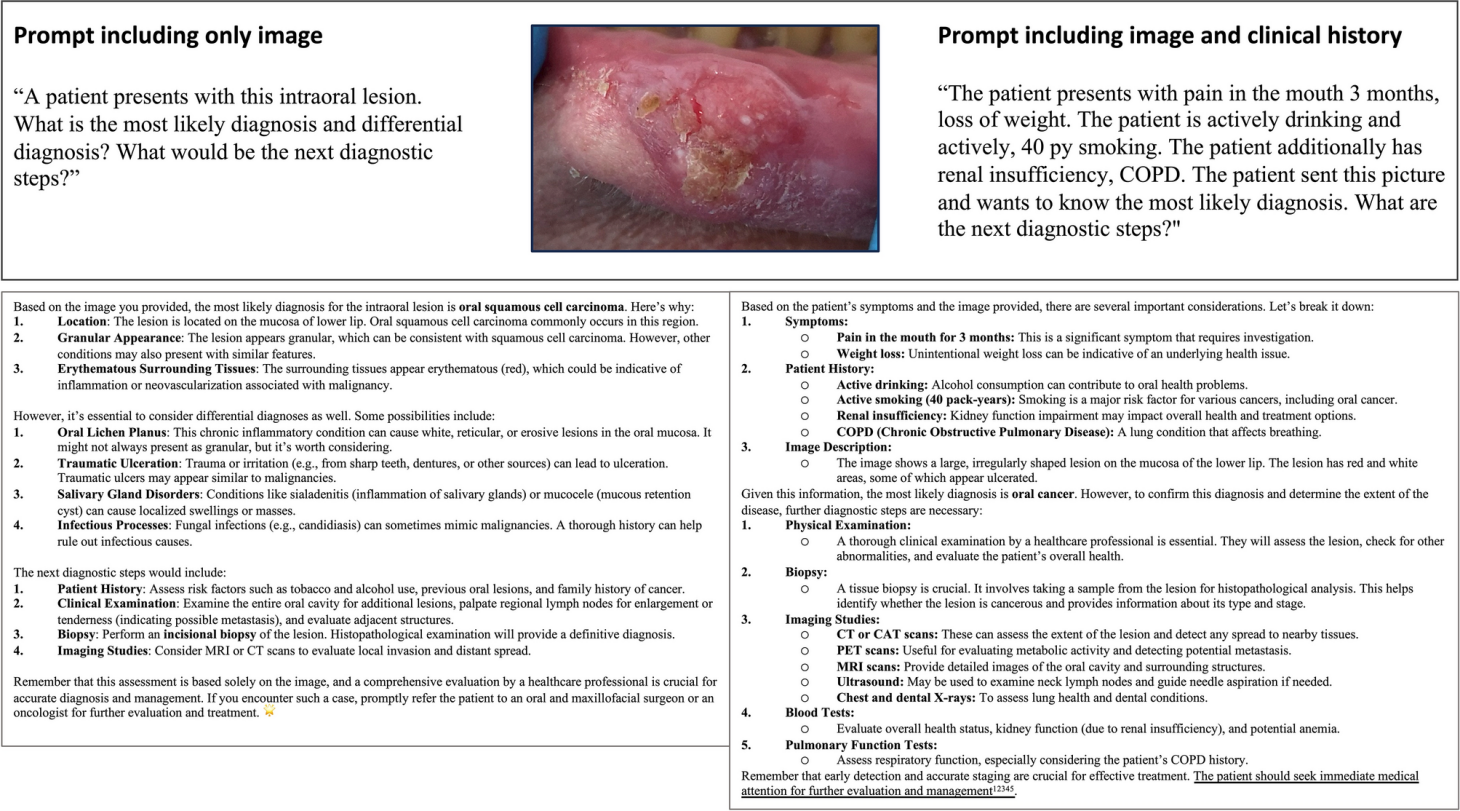
Depiction of an exemplary Oral cancer case and the image. The prompt and the corresponding responses by ChatGPT 4.0, using both prompts are shown. The answers were copied from the response page.

ChatGPT 4.0 responded to all prompts in this study in an exceptionally fast manner, regardless of whether using only an image or the combination of an image and the clinical history of a patient. It consistently recognized that the image used in the prompt is showing the oral cavity or oropharynx and was in most cases able to analyze the image and specify the lesion in more detail including the location and appearance. One example of this is depicted in Fig. 2. In the second scenario of this exploratory study, when the clinical history of a patient was added to the image and the prompt, ChatGPT did not describe the image in the majority of cases but preferred to analyze and dissect the clinical history of the patient. Therefore, the category of “image description” was added to the AIPI to qualitatively rate the ability of image recognition. The modified version of the AIPI additionally rated the performance of listing differential diagnoses (Question 4), the primary diagnosis (Question 5), additional examinations (Question 6) and if these are necessary/useful (Question 7) and if there is a prioritization of the diagnostic steps (Question 8). The most common differential diagnoses generated by ChatGPT for the lesions of the image were oral cancer, oral ulcers, infections: Fungal, viral, or bacterial infection, trauma, and other lesions including benign growths, cysts, and when the clinical history include pain, neuralgia. For Question 4 ChatGPT listed these differential diagnoses of the depicted lesion when using images of SCC, leukoplakia and no lesion. ChatGPT performed best with images of leukoplakia in this scenario. The same could be observed for questions 5 (primary diagnosis) and 7 (additional examinations) with images of leukoplakia reaching the highest performance score. A different result was obtained when the clinical history of the patient was added to the prompt. This improved the performance for the prompts with images of SCCs in Questions 5 and 7, while decreasing the performance of the category “image description”. The performance score of all lesions in the category of image description was reduced when the clinical history was added. Notably, with clinical history included, ChatGPT often omitted reference to the image altogether. When analyzing the results of the prompts without an image, the results were similar to scenario 2 when providing an image and the clinical history. The category of image was not answered by the reviewers since no image was provided. In most cases, ChatGPT referred patients to an otolaryngologist (ENT specialist) or an oral surgeon or specialist highlighting the need for an evaluation by a medical professional. ChatGPT recommended a diagnostic biopsy for definitive histology in almost all cases of a mucosal lesion, while imaging was considered optional for most cases. Exemplary prompts and the corresponding answers generated by ChatGPT 4.0 are depicted in Fig. 2.

In the first scenario, where only an image of the lesion was provided a prompt, only 26% of the images of squamous cell carcinoma were identified by ChatGPT 4.0, whereas 86.7% of leukoplakia images were identified. The images without a lesion were either categorized as benign/no lesion or misclassified as leukoplakia. In the second scenario, when the image was accompanied by the clinical history of a patient, the diagnostic accuracy improved. 73.3% of malignant lesions were accurately detected, whereas 93.3% of benign and 93.3% of the images of an oral leukoplakia were recognized by ChatGPT 4.0.

In addition, when analyzing the results for lesions of the oropharynx compared to lesions of the oral cavity, there was a more accurate diagnosis for images of oral cavity lesions with 26.7% of cases (4 out of 15 cases) diagnosed correctly, while none of the two oropharyngeal lesions were detected. When the clinical history was added, one lesion of the oropharynx, and 73.3% (11 out of 15 cases) of the oral cancer lesions were detected. Furthermore, an analysis of tumor size (T stage) revealed a trend: early-stage SCCs were less likely to be recognized. However, the addition of clinical history consistently improved the recognition rate across stages. When images without a lesion were presented to ChatGPT, it was sometimes stated that there is no lesion, or that there is a lesion that is benign, such as a fissured tongue. In 5 cases (33.3%) of scenario 1 ChatGPT misclassified the image to depict a leukoplakia. When adding the clinical history, this rate reduced to 6.7% (1 out of 15 cases). In the third scenario (clinical history-only), ChatGPT demonstrated a similar performance in detecting SCC (73.3%) as in the scenario 2 (image and clinical history), whereas the performance for leukoplakia was decreased (80%). With only the clinical history as input, ChatGPT suspected leukoplakia or a malignancy in 80% of the benign/negative cases. Notably, when stratified by tumor stage, the model performed better for advanced SCC (T3/T4) compared to early-stage lesions (T1/T2).

**Fig. 3:**
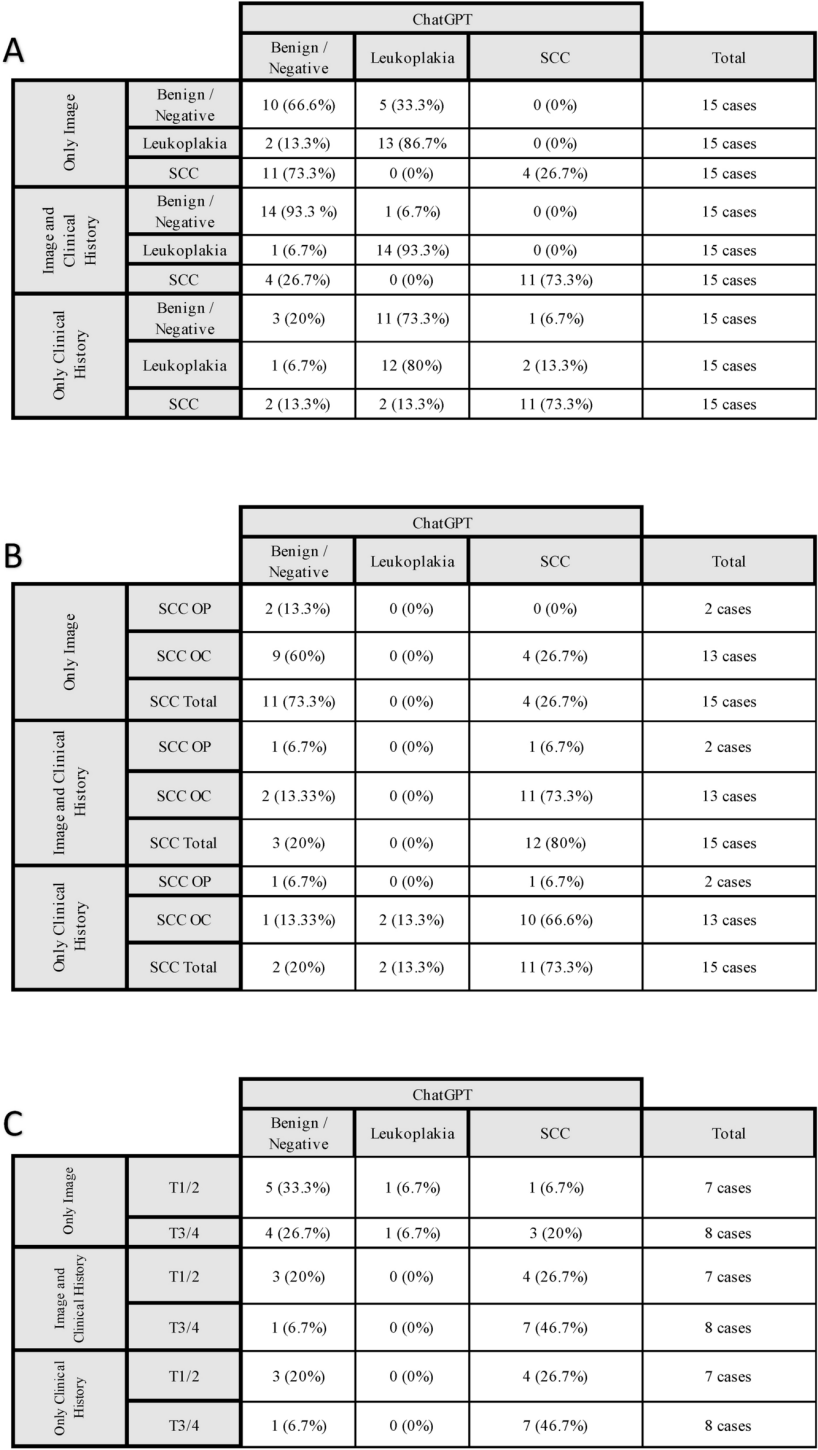
Performance of ChatGPT 4.0 in identifying benign/negative, leukoplakia, and squamous cell carcinoma (SCC) cases based on different scenarios: image-only, image and clinical history, and clinical history only; (**A**) The overall performance of ChatGPT 4.0; (**B**) The results are further stratified by lesion location (oral cavity [OC] vs. oropharynx [OP]) and (**C**) tumor stage (early stage [T1/T2] vs. advanced stage [T3/T4]). Each table summarizes the diagnostic outcomes and highlights the impact of clinical history and lesion characteristics on the accuracy of ChatGPT 4.0’s predictions.

A comprehensive overview of these findings and sub-analyses is presented in Fig. 3.

**Fig. 4:**
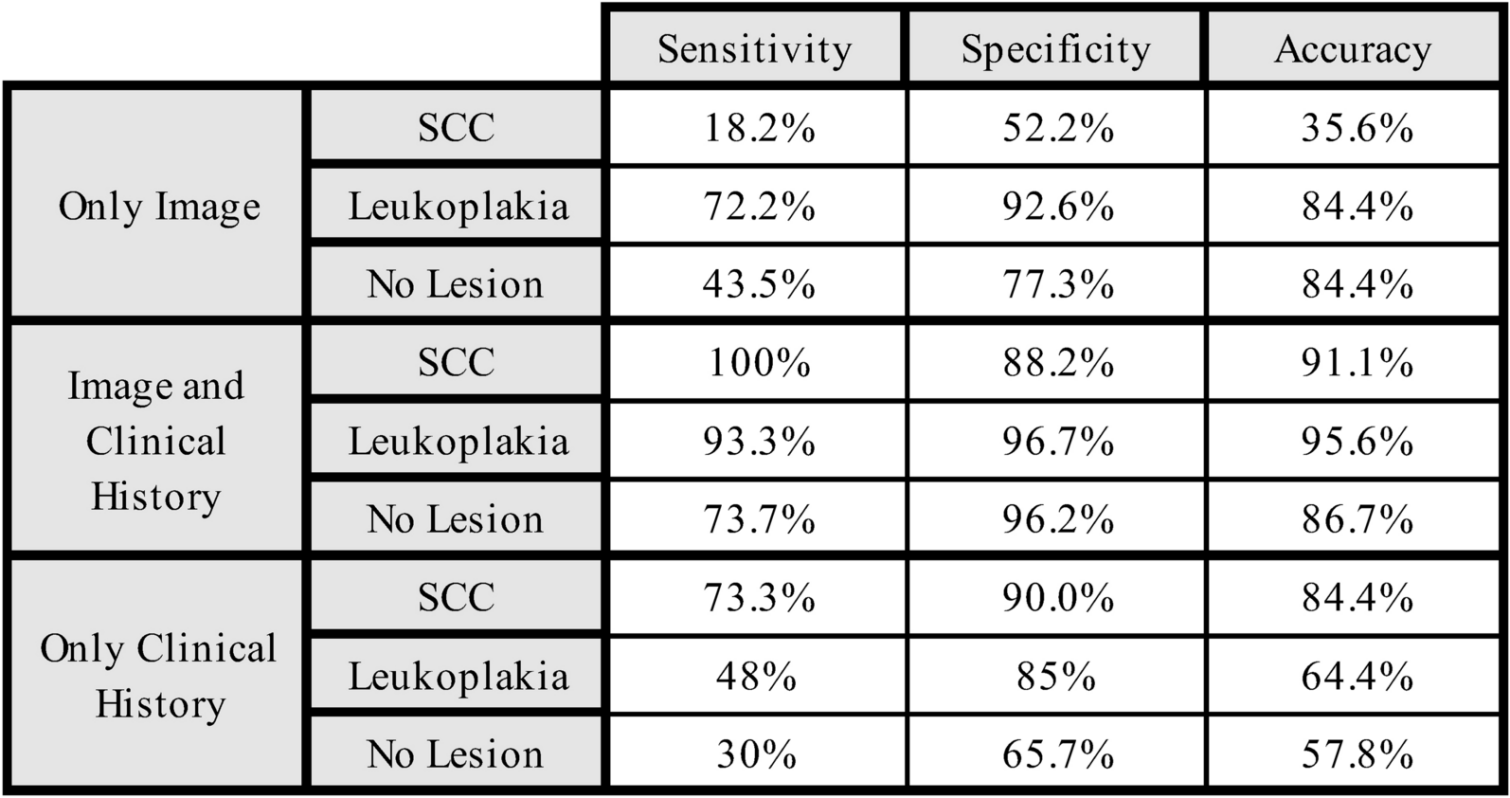
Sensitivity, specificity, and accuracy of ChatGPT 4.0 in diagnosing squamous cell carcinoma (SCC), leukoplakia, and no lesion across three scenarios: (1) Only image, (2) Image combined with clinical history, and (3) Only clinical history.

The sensitivity, specificity, and accuracy of image recognition by ChatGPT of the lesions were calculated and is depicted in Fig. 4. Incorporating the clinical history increased the sensitivity of detecting a squamous cell carcinoma from 18.2 to 100%, while the specificity increased from 52.2 to 88.2%. For leukoplakia the initial sensitivity was 72.2% and specificity was 92.6% and increased to 93.3% and 96.7%. In contrast, images with no lesion exhibited markedly lower sensitivity and specificity compared to leukoplakia and SCC, even when clinical history was included. When only the clinical history was presented, the model achieved a sensitivity of 73.3% for SCC, whereas the sensitivity for the detection of leukoplakia was decreased to 48%. For images with no lesion the sensitivity was the worst out of all three scenarios (30%) (Fig. 4).

**Fig. 5:**
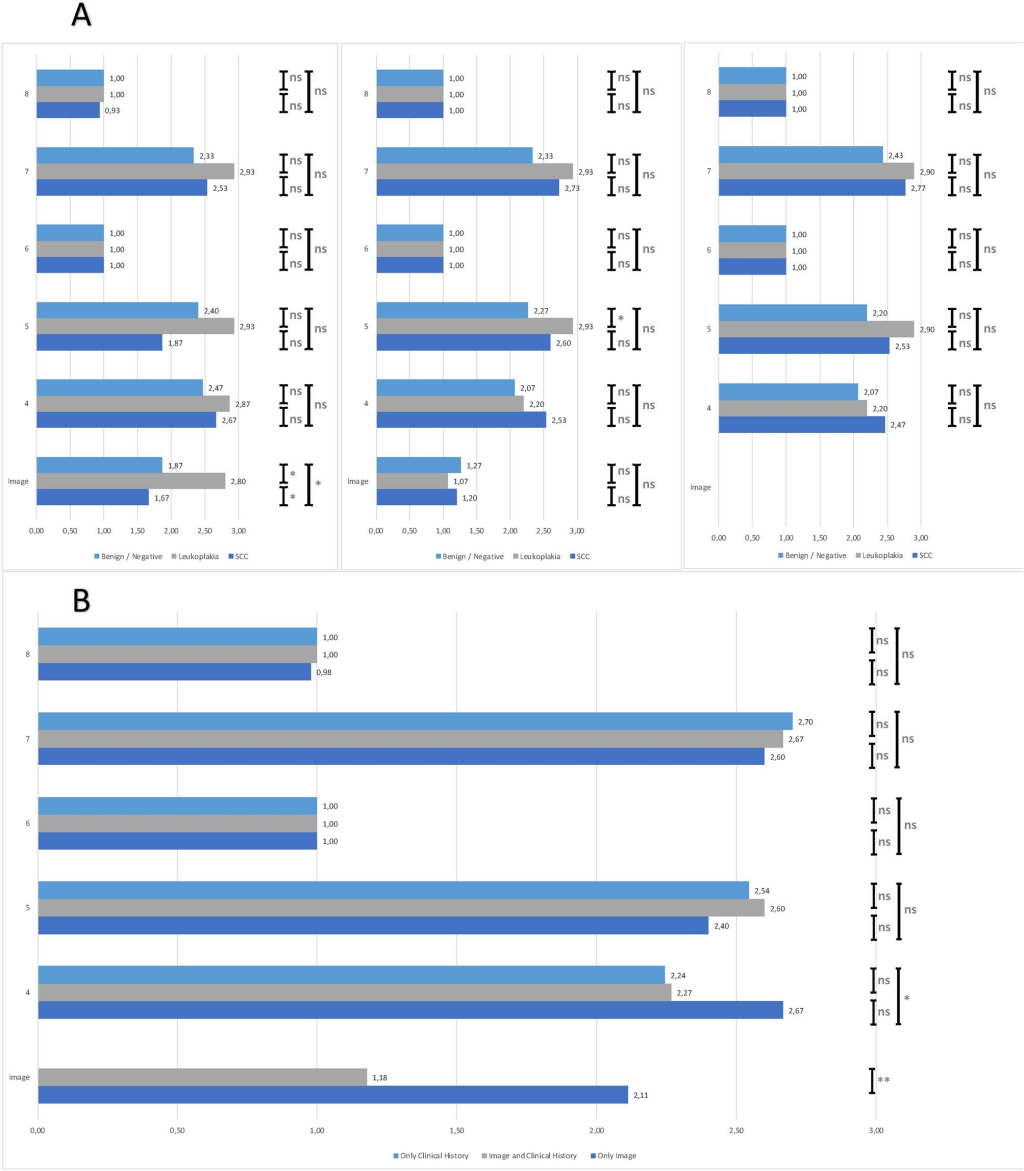
Overall rating of the performance of Image recognition by ChatGPT 4.0. (**A**) Comparison of the grading of the modified AIPI and image description/recognition by two independent reviewers for (a) Scenario 1 with only the image as a prompt; (b) Scenario 2 with the image and the clinical history of each patient; (c) Scenario 3 with only the clinical history, (**B**) Overall comparison of the performance. Each bar is the average of the two independent reviewers grading. * equals *p* > 0.05, ** equals *p* > 0.01, *** equals *p* > 0.001.

All performance ratings generated by the ChatGPT were evaluated by two independent reviewers and inter-rater agreement was assessed using Cohen’s κ. When comparing the results, the reviewers reached an agreement measured by Cohen’s κ of 0.498 for image recognition, 0.529 for question 4, 0.773 for question 5, 1.0 for question 6, 0.287 for question 7, and 0.357 for question 8. In the second scenario, two independent reviewers reached an agreement measured by Cohen’s κ of 0.686 for image recognition, 0.749 for question 4, 0.468 for question 5, 1.0 for question 6, 0.493 for question 7, and 1.0 for question 8. The results of the modified AIPI for each lesion is depicted in Fig. 5.

Each of the 45 cases was analyzed in detail to explore areas of significant expertise in the image recognition in certain clinical scenarios (Supplementary Fig. 1). No clear pattern of areas of specialized expertise could be observed, however, in most cases, Scenario 1 achieved a higher overall score than Scenario 2. Exceptions included cases 6, 12, 13, and 14 of the SCC images, case 5 of the leukoplakia images, and cases 7 and 15 of the images without a lesion.

ChatGPT 4.0 consistently emphasized in its responses that the final diagnosis and clinical decisions should be discussed with a physician. Some of the Limitations of the use of LLMs for image interpretation were already stated by ChatGPT, such as the quality of the provided image and the uniqueness of individual patient scenarios. ChatGPT 4.0 often listed the source material and even linked images that resembled the image of the prompt.

## Discussion

This is the first study evaluating the use of the artificial intelligence-based image recognition feature of ChatGPT 4.0 for the diagnosis of oral cavity and oropharyngeal squamous cell carcinoma, subsets of head and neck squamous cell carcinoma. In this exploratory study 45 intraoral images were used in two different clinical scenarios, once with only the information of the image, and a second time with addition of the clinical history of the patient. The ability to make the right diagnosis, as well as the recommendations of ChatGPT for the diagnostic steps were evaluated by two different reviewers. The aim of this study was to investigate the potential and limitations of image recognition by an advanced LLM and to assess a potential use in the clinical setting.

At the time of this study there are no studies in the literature evaluating this tool for image recognition of head and neck cancer, with only one other exploratory study for the diagnosis of melanoma^20^. Oral cavity and Oropharyngeal Carcinoma were investigated in this study, since the incidence of this malignancy is rising, and the lesions are easily accessed to take an image. These images can be observed by medical professionals from remote but might also be evaluated by artificial intelligence tools. ChatGPT 4.0 is a Natural Language Processing (NLP), a subset of artificial intelligence (AI) dedicated to analyzing human language. The use of LLMs was already investigated in different applications, including medical education, rheumatology, breast cancer research and different medical examinations^18,23–27^.

In this study, ChatGPT 4.0 achieved a sensitivity of 18.2% and specificity of 52.2% for detecting oral cancer when the prompt consisted of only an image and the LLM was only able to analyze the information of the image to find the most likely diagnosis of oral cancer. This result is similar to a study investigating the use of ChatGPT 4.0 for analyzing the images of melanoma, in which a sensitivity of 32.0% and specificity of 40% was achieved^20^. Since in the clinical setting most patients don’t show an image of a lesion, but complain about symptoms, a second scenario was investigated in this study, in which the image was used in prompt, with additional information about the clinical history. In this second scenario, the sensitivity and specificity of ChatGPT increased to 100% and 88.2%. Especially the symptom of pain was often part of the clinical history of the patients in this study and might have led to the diagnosis of oral/oropharyngeal cancer. This was also observed in scenario 3, when only the clinical history was presented, and more advanced cases were also diagnosed more consistently. In scenario 3, providing only the clinical history, a mediocre sensitivity of 70% was achieved for SCC, whereas leukoplakia and cases without a lesion were mostly misclassified, which again suggests the text-dependent nature of LLMs.

ChatGPT 4.0 achieved a remarkable result for the detection of leukoplakia with an already high sensitivity of 72.2% and specificity of 92.6% when getting only the image, and an impressive sensitivity of 93.3% and specificity of 96.7% when the clinical history was included in the prompt. When images without a lesion or with a benign lesion were displayed, five lesions in scenario 1 and one lesion in scenario 2 were recognized as leukoplakia, even though there was no visible lesion in the image. The situation was even more emphasized when only the clinical history and no image was used as the prompt and leukoplakia, or a malignancy was suspected in the majority of cases. In another study^20^ of diagnosing melanoma with ChatGPT, the LLM even classified some images of benign lesions as melanoma. In general, in our study more advanced lesions were recognized more often, and oral cavity lesions more often than oropharyngeal cancer. This is probably due to the difficulty of obtaining sufficient images of the oropharynx, or a lack of images of oropharyngeal lesions in the databases that are the basis of ChatGPT. Unfortunately even though some images and source material of the responses are already displayed, the mechanics and databases that lead to a specific answer of ChatGPT are most of the times unclear, which is often referred to as “black box phenomenon”^28^. This was also observed in other studies^18,23–27^.

LLMs were able to assess and diagnose head and neck cancer based on the written symptoms of patients in former studies and an artificial tumor board reached a high concordance in breast cancer and head and neck cancer. One study investigated fictional cases of patients with various advanced cancer with genetic alterations provided by 4 different LLMs and one physician. The LLMs were able to provide large numbers of different therapeutic options but did not provide any convincing clinical reasoning to support their recommendations and did not reach the level of the expert^29^. This is similar to the high ratings of the performance seen in our study in question 4, in which ChatGPT listed differential diagnoses in a highly effective way. This ability to generate written information based on a database is one of the core strengths of an LLM^18,30^. A modified version of the artificial intelligence performance index (AIPI)^22^ was used to assess the diagnostic performance of ChatGPT in this study. Interestingly the newly added category of image description was significantly better rated by two independent reviewers, when only an image was used as prompt. This scenario also achieved a better rating for stating the differential diagnoses for lesion of the oral cavity. When comparing the modified AIPI rating of squamous cell carcinoma, leukoplakia and images without a lesion, leukoplakia achieves high ratings in both scenarios, while SCC is inferior in scenario 1. While there are already numerous tools for the evaluation of skin cancer by digital dermoscopy images as a backup for specialist diagnosis to assist in minimizing the risk of missing melanomas^31^, there are only a few studies investigating the use of artificial intelligence tools for the diagnosis of oral cavity/oropharyngeal squamous cell carcinoma^32^. Most of these approaches were highly technical including Nayak et al. who used Machine Learning to discriminate oral lesions between normal, premalignant, and tissues using laser-induced autofluorescence spectra recordings and reached an accuracy of 98.3%, specificity of 100%, and sensitivity of 96.5%^33^. Studies on histopathological images found an accuracy in a range from 89.47 to 100%, sensitivity from 97.76 to 99.26%, and specificity ranging from 92 to 99.42% in a recent review^34^. Since ChatGPT is currently not able to analyze histopathological slides, it is difficult to compare the results of this study. There are a few elegant population-based studies in India, in which a mobile phone was connected with an intraoral probe to make a point-of-care screening tool for oral cancer including neural networks^8,35^. This approach achieved an accuracy of 81%, a sensitivity of 85% and a specificity of 84%, clearly surpassing the results of our study when only an image was used as the prompt. In our study the additional information of the clinical history could achieve a higher sensitivity and specificity. This may be due to the fact that LLMs are by nature text dependent and might prefer text-based information. This observation cannot be backed up by data in the literature yet but might explain why ChatGPT did not mention the image in many cases of this study when the clinical history was also available.

While the results of these studies and recent developments in artificial intelligence-based image recognition warrant future research in this promising field, there are still many limitations before implementation of artificial intelligence into the clinical practice. Firstly, the lack of transparency of the resources used to generate the answer^28^. While guidelines and clinical studies are the backbone of clinical decision making, the access to the source information of LLMs is still very limited. Validation and reproduction are therefore highly limited as reported by many studies^10,36,37^. Although this exploratory study analyzed the currently largest number of images of oral/oropharyngeal lesions and this is the first time the image recognition feature of ChatGPT 4.0 was evaluated for this purpose, there might be a level of heterogeneity of the patients’ images, which influenced the results of this study. Another limitation of the use of an LLM is the importance of the prompt design^15,25,29^. Variations in the prompt lead to different responses by LLMs and influence the results of any study. In the preparation of this study 8 different prompts were tested, and two different prompts, resulting in two different clinical scenarios, one with only an image as the prompt and another one with an image and the clinical history of the patient were used. This monocentric study investigated the images of the patients of only one European institution, which is a limitation due to analyzing only images of Caucasian patients. In many studies it has been demonstrated that there are differences when analyzing skin cancer in different ethnic groups^38^, and LLMs can adjust to practices and changes in different regions and the source and diversity of training data^39^. Future studies might benefit from a multicentric/multiethnic approach, with the accessibility of LLMs as one of the major benefits of LLMs. Simultaneously, the use of LLMs for image interpretation in clinical settings raises significant ethical concerns. A major limitation is the potential for misdiagnosis, as LLMs can provide incorrect or inconsistent interpretations, as seen in the sensitivity and specificity in this study, which might endanger patient safety. Privacy and data security are also critical, as using clinical images might contain sensitive patient information. In addition, LLMs might be biased if the training data lacks diversity or contains systemic inaccuracies. Unfortunately, the source data is not publicly available at the moment^40,41^. Addressing these ethical concerns requires careful validation and improvements in terms of data transparency before integration of LLMs into clinical workflows.

Currently LLMs are not programmed to think independently but generate a text-based output based on public documents and databases^10^. While in the clinical setting the otolaryngologist/maxillofacial surgeon/dentist can palpate the lesion, or ask additional questions about the clinical history, or make another image from a slightly different angle, an LLM is not able to “think outside the box”. Additionally, while the treatment of HNSCC remains a complex field due to the heterogenous nature of the disease, even the appearance of oral lesions is highly heterogeneous. A study investigating a larger number of images of oral lesions might lead to better results when only an image is used as a prompt, but the combination of the clinical history and an image led to a significantly better sensitivity and specificity with the current version of ChatGPT 4.0 and therefore is more suitable for implementation into the clinical setting. At the moment the diagnosis of lesions of the oral cavity and oropharynx by ChatGPT needs to be evaluated carefully by medical professionals based on their clinical knowledge and cannot be used as a clinical guidance. This is also addressed by the LLMs themselves at the end of most of the responses in this study.

## Conclusions

The study is the first highlighting the potential of artificial intelligence-based image recognition using ChatGPT 4.0 for assessing clinical images of oral and oropharyngeal squamous cell carcinoma and leukoplakia. The findings highlight that, at present, combining image analysis with clinical history is important to achieve convincing diagnostic accuracy, as image recognition alone remains limited in sensitivity and specificity. Notably, leukoplakia images demonstrated high sensitivity and specificity without requiring additional clinical history. These results suggest that artificial intelligence could play a significant role in the future clinical evaluation of early stages of cancer and oral lesions, paving the way for more accessible and efficient diagnostic tools.

## Supplementary Information

Supplementary Information.

## Data Availability

Data is provided within the manuscript or supplementary information files. The datasets generated and/or analysed during the current study are available in the Supplementary Material.
