# Supplementary material for: Artificial intelligence for image recognition in diagnosing oral and oropharyngeal cancer and leukoplakia
Source: Sci Rep. 2025 Jan 29;15:3625. doi: 10.1038/s41598-025-85920-4 (PMC11779835; doi:10.1038/s41598-025-85920-4)
Supplement: Supplementary file 1 — Supplementary Information. [file 41598_2025_85920_MOESM1_ESM.docx]

(c)

(b)

(a)

(d)

**Supp. Figure 1. Rating of the performance of ChatGPT 4.0 for each of the 45 cases**. Comparison of the sum of Image recognition and modified AIPI grading by two independent reviewers. (a) Overview of the overall results; (b) Results of the SCC images; (c) Results of leukoplakia images; (c) Results of images without a lesion. Each bar is the average of the two independent reviewers grading

|  | Monday | | | Wednesday | | | Friday | | |
| --- | --- | --- | --- | --- | --- | --- | --- | --- | --- |
| Browser 1 | Case 3 | Case 24 | Case 42 | Case 3 | Case 24 | Case 42 | Case 3 | Case 24 | Case 42 |
| Original Suspicion | SCC | Leukoplakia | Leukoplakia | SCC | Leukoplakia | Leukoplakia | SCC | Leukoplakia | Leukoplakia |
| Morning (8 AM) | 3/3 | 3/3 | 3/3 | 3/3 | 3/3 | 3/3 | 3/3 | 3/3 | 3/3 |
| Afternoon (3 PM) | 3/3 | 3/3 | 3/3 | 3/3 | 3/3 | 3/3 | 3/3 | 2/3 | 3/3 |
| Evening (9 PM) | 3/3 | 3/3 | 2/3 | 3/3 | 3/3 | 3/3 | 3/3 | 3/3 | 3/3 |
| Browser 2 | Case 3 | Case 24 | Case 42 | Case 3 | Case 24 | Case 42 | Case 3 | Case 24 | Case 42 |
| Original Suspicion | SCC | Leukoplakia | Leukoplakia | SCC | Leukoplakia | Leukoplakia | SCC | Leukoplakia | Leukoplakia |
| Morning (8 AM) | 3/3 | 2/3 | 3/3 | 3/3 | 3/3 | 3/3 | 2/3 | 3/3 | 3/3 |
| Afternoon (3 PM) | 3/3 | 3/3 | 3/3 | 3/3 | 3/3 | 3/3 | 3/3 | 3/3 | 3/3 |
| Evening (9 PM) | 3/3 | 3/3 | 3/3 | 3/3 | 3/3 | 3/3 | 3/3 | 3/3 | 3/3 |
| Browser 3 | Case 3 | Case 24 | Case 42 | Case 3 | Case 24 | Case 42 | Case 3 | Case 24 | Case 42 |
| Original Suspicion | SCC | Leukoplakia | Leukoplakia | SCC | Leukoplakia | Leukoplakia | SCC | Leukoplakia | Leukoplakia |
| Morning (8 AM) | 3/3 | 3/3 | 3/3 | 3/3 | 3/3 | 3/3 | 3/3 | 3/3 | 3/3 |
| Afternoon (3 PM) | 3/3 | 3/3 | 3/3 | 3/3 | 3/3 | 3/3 | 3/3 | 3/3 | 3/3 |
| Evening (9 PM) | 3/3 | 3/3 | 3/3 | 3/3 | 3/3 | 2/3 | 3/3 | 3/3 | 2/3 |

**Supp. Figure 2. Rating of the consistency of ChatGPT 4.0 for three random cases (3, 24 and 42) using three different web browsers on three different days and three different times of the day to generate a response**. Comparison of the original suspicion, such as SCC in case 3 and the response in the consecutive runs to test constancy in the three runs on each time point and browser, e.g. 3/3 equals the suspicion of SCC in all three runs.

**Supp. Table 1. Clinical and pathological data of the patient cohort. There were 45 patients included in this study.** In addition, the answers of ChatGPT when asked with only an image, or the combination of the image and the clinical history, or only the clinical history was added. Abbreviations: NA = not available, OC = Oral Cavity, OP = Oropharynx.
